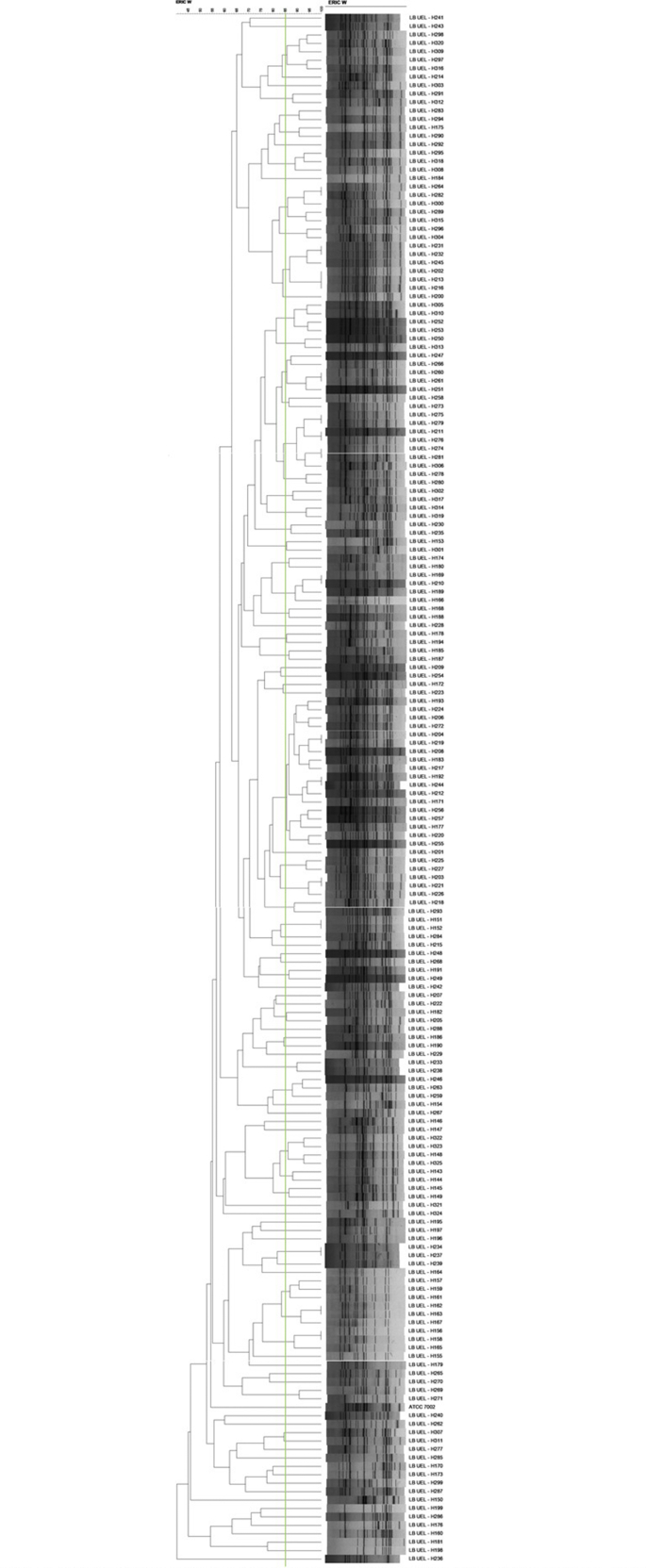# Virulence, resistance and clonality of *Proteus mirabilis* isolated from patients with community-acquired urinary tract infection (CA-UTI) in Brazil

**DOI:** 10.1016/j.micpath.2020.104642

**Published:** 2021-03

**Authors:** Wellington Danilo de Oliveira, Mário Gabriel Lopes Barboza, Gabriela Faustino, Willian Teruhiko Yamanaka Inagaki, Matheus Silva Sanches, Renata Katsuko Takayama Kobayashi, Eliana Carolina Vespero, Sergio Paulo Dejato Rocha

**Affiliations:** a Laboratory of Bacteriology, Department of Microbiology, Center of Biological Sciences, State University of Londrina (UEL), Londrina, PR, Brazil; b Laboratory of Basic and Applied Bacteriology, Department of Microbiology, Center of Biological Sciences, State University of Londrina (UEL), Londrina, PR, Brazil; c Department of Pathology and Clinical and Toxicological Analysis, State University of Londrina (UEL), Londrina, PR, Brazil

**Keywords:** Bacterial pathogenesis, Virulence genes, Biofilm, Clonal relationship, Antimicrobial resistance, Urinary infection

## Abstract

Urinary tract infections (UTIs) are among the most common human infections, both in hospitals and in communities. *Proteus mirabilis* is known to cause community-acquired urinary tract infection (CA-UTI) and is an important causative agent of nosocomial UTIs. The pathogenesis of this species is related to its ability to manifest virulence factors, such as biofilms, adhesion molecules, urease, proteases, siderophores, and toxins. In this study, we investigated the virulence, sensitivity to antimicrobials, and clonal relationship of 183 strains isolated from the urine of CA-UTI patients in Londrina, Paraná State, Brazil. A total of 100% of the strains were positive for *hpmA, ptA, zapA, mrpA, pmfA, ireA, and atfA* virulence genes. The *ucaA* gene was positive in 81.4% of the cases. The strains showed high rates of sensitivity to the evaluated antimicrobials, and only one was ESBL-positive. All the tested bacteria showed the capacity to form biofilms: 73.2% had a very strong intensity, while 25.7% had a strong intensity, and 1.1% had a moderate intensity. Regarding clonality, 40 clonal clusters were found among the microorganisms tested. Our results showed that strains of *P. mirabilis* isolated from CA-UTI patients have several virulence factors. Although the urinary clinical isolates studied showed high sensitivity to antimicrobials, the strains showed a strong capacity to form biofilms, making antibiotic therapy difficult. In addition, it was observed that there were clones of *P. mirabilis* circulating in the city of Londrina.

## Highlights

• All strains presented a variety of virulence genes.
• It was observed that there were clones of *P. mirabilis* circulating.
• 98.1% of strains produced strong or very strong biofilm.

## 1 Introduction

Urinary tract infections (UTIs) are one of the most common human infections, occurring both in hospitals and in communities (CA-UTI) [32]. These infections can affect any part of the urinary system (including the kidneys, ureters, bladder, and urethra) and it is estimated that approximately 150 million people are affected by UTIs worldwide, each year (12,34).

*Proteus mirabilis* is known to cause CA-UTI and is an important agent of nosocomial UTIs [2]. *P. mirabilis* expresses several virulence factors involved in the pathophysiology of UTIs, including biofilm formation, production of enzymes and cytotoxins, motility, and iron acquisition systems [1].

Normally, *P. mirabilis* is susceptible to beta-lactam antibiotics, aminoglycosides, fluoroquinolones, and trimethoprim-sulfamethoxazole, but has intrinsic resistance to polymyxin, nitrofurantoin, and tetracycline [8]. The empirical prescription of antimicrobials for CA-UTI is common practice; however, bacterial resistance to antimicrobials has been increasing globally, which represents a decrease in the effectiveness of empirical treatment [29].

Information on the prevalence of *P. mirabilis* causing CA-UTIs, as well as on virulence, resistance to antimicrobials, and clonality, is rare. Therefore, the objective of the present study was to evaluate the virulence markers, resistance patterns, and clonal distribution of *P. mirabilis* isolated from the urine of CA-UTI patients in the city of Londrina, Brazil.

## 2 Materials and methods

### 2.1 Bacterial strains and identification

A total of 183 isolates of *P. mirabilis* were studied. These microorganisms were isolated from urine cultures of CA-UTI patients, assisted at 44 Basic Health Units (BHU) in Londrina, Brazil. Urine samples were collected from December 2016 to July 2017. The bacterial isolates were identified using the Vitek® 2 Compact system (BioMérieux, MarcyL’Etoile, France). Only urine samples with ≥10^5^ colony forming units (CFU) were selected for the study. The microorganisms identified were named LBUEL–H143 to LBUEL–H325. This research was approved by the Research Ethics Committee (CEP-1.590.120).

### 2.2 Detection of antimicrobial resistance

The antimicrobial resistance profile of the 183 *P. mirabilis* isolates was determined by using the automated Vitek® 2 Compact system (BioMérieux, MarcyL'Etoile, France) and by following the interpretation of the Clinical and Laboratory Standards Institute (CLSI) [10]. The following antimicrobials were tested: nalidixic acid, amikacin, amoxicillin + clavulanic acid, ampicillin, cephalothin, cefepime, ceftriaxone, cefuroxime, ciprofloxacin, ertapenem, gentamicin, meropenem, nitrofurantoin, norfloxacin, piperacillin + tazobactam, and trimethoprim + sulfamethoxazole. Isolates resistant to cephalosporins of the 3rd and 4th generations were subjected to double disc synergy test in order to evaluate the phenotypic production of ESBL (extended-spectrum beta-lactamases), using the antimicrobials recommended by the Clinical and Laboratory Standards Institute (CLSI) [9]. Using polymerase chain reaction (PCR), ESBL genes from CTX-M (1, 2, 8, 9, and 25) [33] and AmpC families were evaluated (*MOX*, *FOX*, *EBC*, *AAC*, *DHA*, *CIT*, and *CMY-2*) [26].

In addition, the isolates that showed resistance to other classes of antimicrobials were submitted to PCR to search for the following resistance genes: *qnrA, qnrB, qnrS* [5], *qnrD* [6] - quinolones; aac (6′) - Ib-cr [9] - quinolones/aminoglycosides; *sul1* and *sul2* [19] - sulfonamides.

### 2.3 Detection of virulence genes

The detection of virulence genes was performed by using PCR. The following genes were analyzed: *ptA, zapA, ucaA, ireA* [30], *hpmA* [7], *mrpA* [28], *pmfA* [36], and *atfA* [35]. The bacterial DNA was extracted using the boiling method, followed by thermal shock. PCR was performed in a thermocycler containing a final volume of 25 μL, which was composed of 1.0 μL of MgCl_2_ - 2 mM - (Invitrogen®), 2.5 μL of 10X buffer (Invitrogen®), 2.5 μL of dNTPs - 0.2 mM (Invitrogen®), 0.5 μL of Forward Primer - 20 pmol, and 0.5 μL of Reverse Primer - 20 pmol (Invitrogen®), 0.5 μL of Taq DNA polymerase - 1.5 U/μL (Invitrogen®), 2.0 μL of bacterial DNA, and 15.5 μL of ultrapure water. The standard *P. mirabilis* HI4320 strain was used as a positive control and the reaction without DNA was used as a negative control. After the PCR, the samples were subjected to electrophoresis in an agarose gel at a concentration of 1%–2%, according to the size of the fragment to be amplified, which was stained with SYBR SAFE (Invitrogen®) and emerged in Tris-Borate-EDTA buffer (TBE) (89 mM Tris base, 89 mM Boric Acid, 2 mM EDTA; pH 8.3). The molecular marker used was a 1 kb ladder (Invitrogen®). The system was subjected to a constant voltage of 70 V for 50 min. After the estimated time, the gel was observed using a transilluminator with an ultraviolet light.

### 2.4 Biofilm formation assay

The determination of biofilm formation was carried out in 96-well polystyrene plates using crystal violet, according to Ref. [18]. The standard *E. coli* EAEC-042 strain was used as a positive control for biofilm formation, and trypticase soy broth (TSB) (Difco) was used as a negative control. The absorbance (A) was measured on a spectrophotometer at a wavelength of 570 nm.

### 2.5 Analysis of the genetic similarity profile

The genetic similarity profile between the strains was determined using the enterobacterial repetitive intergenic consensus (ERIC-PCR) technique. The following primers were used: ERIC-1R (5′-ATGTAAGCTCCTGGGGATTCAC-3′) and ERIC-2 (5′-AAGTAAGTGACTGGGGTGAGCG-3′), according to Ref. [21]. Each reaction was performed containing using a final volume of 25 μL, composed of 1.0 μL of MgCl_2_ - 2 mM - (Invitrogen®), 2.5 μL of 10X buffer (Invitrogen®), 2.5 μL of dNTPs - 0.2 mM (Invitrogen®), 0.5 μL of Forward Primer - 20 pmol and 0.5 μL of Reverse Primer Reverse - 20 pmol (Invitrogen®), 0.5 μL of Taq DNA polymerase - 1.5 U/μL (Invitrogen®), 2.0 μL of bacterial lysate, and 15.5 μL of ultrapure water. For DNA amplification, each sample was subjected to an initial denaturation step for 4 min at 92 °C, followed by 40 denaturation cycles of 1 min at 94 °C, annealing for 1 min at 48 °C, an extension for 5 min at 72 °C and a final extension at 72 °C for 5 min. After amplification, the samples were subjected to 1.5% agarose gel electrophoresis and stained with ethidium bromide (Invitrogen®). All samples were performed in triplicates to confirm the reproducibility of the band profiles obtained. A similarity dendrogram was constructed using BioNumerics v. 5.1 (AppliedMaths®, Keistraat, Belgium) using the unweighted pair group method with arithmetic mean (UPGMA) method and the dice similarity coefficient for the analysis of the clusters. Only bands between 200 bp and 3000 bp were included in the analyses. The standard cutoff level to define the clusters was 85%, since this is the standard cutoff for Gram-negative bacteria in a previous study with epidemiological purposes [14].

### *2.6* S*tatistical analysis*

Statistical analysis was performed using the statistical software R version 3.6.3, with a 95% confidence interval (CI), and the results were considered significant when the p value was <0.05.

## 3 Results

A total of 183 strains of uropathogenic *P. mirabilis* were evaluated in this study; 146 were isolated from females (79.7%) and 37 (20.2%) from males. The age of the patients ranged from 1 to 95 years. Forty-six (25.1%), 15 (8.2%), 75 (40.9%), and 47 (25.6%) strains were obtained from patients aged 0–12; 13–18, 19–59, and 60–95 years, respectively. Of the 146 female patients, 34 (18.5%) were pregnant. All urinary isolates were collected in 44 different BHUs (named BHU-01 to BHU-44), of which BHU-22 was the most prevalent, with 13 isolates.

Statistical analyses showed that CA-UTI by *P. mirabilis* is 182.58 times more likely to occur in women aged 16–31 years (p < 0.001/CI: 0-infinite), and 122.6 times more likely to occur in women aged 32–47 years (p = 0.006/CI: 0-infinite). In men, CA-UTI by *P. mirabilis* was 5.15 times more likely to occur in the age group from 48 to 62 years (p = 0.009/CI: 1.44–18.45).

In the evaluation of the antimicrobial sensitivity profiles, the following results were found: 78.1%, 94.5%, 96.7%, 99.5%, 80.3%, 97.8%, 98.4%, 100% of the bacterial isolates were sensitive to trimethoprim + sulfatomexazole; nalidixic acid and gentamicin; norfloxacin and ciprofloxacin; amikacin and amoxicillin + clavulanic acid; ampicillin; cephalothin; cefuroxime, ceftriaxone, and cefepime; and ertapenem, meropenem, piperacillin + tazobactam, respectively.

Trimethoprim + sulfatomexazole was the antibiotic combination with the highest resistance rate, followed by ampicillin. Resistance to trimethoprim + sulfamethoxazole was not an important factor between the age groups and genders of the patients (p > 0.05).

According to Table 1, 15 different resistance patterns were found among the tested isolates. Of the total number of microorganisms studied, 135 (73.8%) were not resistant to the antibiotics evaluated, while 15 (8.1%) were resistant to one class of antibiotics, 20 (10.9%) were resistant to two classes of antibiotics, and 13 (7.1%) presented a multidrug resistance phenotype (resistant to, at least, three different classes of antibiotics). Only the LBUEL–H301 isolate was positive for the ESBL production phenotype.

**Table 1:** Antimicrobial resistance patterns.

Resistance Pattern (RP)	Antimicrobials	Total number of strains
RP 01	Sensible	135
RP 02	NAL	1
RP 03	AMP	4
RP 04	SXT	10
RP 05	AMP, GEN	2
RP 06	AMP, SXT	18
RP 07	AMP, GEN, SXT	2
RP 08	AMP, SXT, NAL	2
RP 09	AMC, AMP, CEP, GEN	1
RP 10	CIP, GEN, NOR, SXT, NAL	1
RP 11	AMP, CIP, NOR, SXT, NAL	2
RP 12	AMP, CIP, GEN, NOR, SXT, NAL	2
RP 13	AMP, CEP, FEP, CRO, CXM, GEN, SXT, NAL	1
RP 14	AMI, AMP, CEP, FEP, CRO, CXM, GEN, SXT	1
RP 15	AMP, CEP, FEP, CRO, CXM, CIP, NOR, SXT, NAL	1

AMC: amoxicillin + clavulanic acid; AMI: amikacin; AMP: ampicillin; CEP: cephalothin; CIP: ciprofloxacin; CRO: ceftriaxone; CXM: cefuroxime; FEP: cefepime; GEN: gentamycin; NAL: nalidixic acid; NOR: norfloxacin; SXT: trimethoprim + sulfamethoxazole.

Bacterial strains that presented a resistance phenotype to, at least, one antimicrobial class among quinolones, sulfonamides, and/or aminoglycosides, were submitted to PCR to evaluate the presence of some resistance genes. The *qnrA, qnrB, qnrD, qnrS*, and aac (6’)-Ib-cr genes were not found in the tested microorganisms. Of the total of 40 strains that were resistant to trimethoprim + sulfamethoxazole, 7 (17.5%) were positive for *sul1* and 20 (50%) were positive for *sul2*; 4 (10%) strains were positive for both genes. The genes of the families AmpC (*MOX*, *FOX*, *EBC*, *AAC*, *DHA*, *CIT*, and *CMY-2*) and CTX-M (1, 2, 8, and 25) were not found in any of the strains that presented resistance to 3rd and 4th generation cephalosporins. The CTX-M-9 gene was positive for the LBUEL-H301 strain (the only strain having an ESBL production phenotype).

Regarding virulence genes, the following genes were searched: *hpmA, ptA, zapA, mrpA, pmfA, ucaA, ireA,* and *atfA*. Of the total strains studied, 149 (81.4%) were positive for all genes, while 34 (18.5%) were positive for all genes, except for *ucaA* gene. There was no statistically significant difference between the presence of the *ucaA* gene and resistance to the evaluated antimicrobials (p > 0.05).

In this study, all strains tested were capable of forming biofilms. Of the 183 strains, 134 (73.2%) had a very strong biofilm formation capacity, 47 (25.6%) had a strong capacity, and 2 (1.1%) had a moderate capacity to form biofilms. Strains producing moderate biofilms were isolated from female patients only and from the following age groups: 0–12 and 19–59 years. Strains with a strong and very strong biofilm formation capacity were isolated from both sexes and all age groups.

Considering the intensity of biofilm formation and the resistance to antimicrobials, the prevalence of resistance to ampicillin was higher in strains that had a strong intensity (27.1%) than in those that had a very strong intensity (17.2%). The same was observed with other antimicrobials: ciprofloxacin (8.5% of strains had a strong intensity and 1.5% had a very strong intensity); gentamicin (10.6% of strains had a strong intensity and 3.7% had a very strong intensity), norfloxacin (8.5% of strains had a strong intensity and 1.5% had a very strong intensity); trimethoprim + sulfatomexazole (25.5% had a strong intensity and 20.9% had a very strong intensity); and nalidixic acid (12.8% had a strong intensity and 2.2% had a very strong intensity). There was no significant difference between the intensity of biofilm formation and the age and gender of the patients (p > 0.05).

Regarding clonal diversity, 40 different clonal cluster (CC) were found (Table 2 and Fig. S1). The number of strains belonging to each cluster varied between 2 and 18. We found 15 clones (31 strains) with 100% similarity between them (Table 3). Of these, three were the same clones (LBUEL-H202, LBUEL-H213, and LBUEL-H216). In clonal cluster 8 and 24, two distinct clones were found, while in clonal cluster 13, three clones were found. In clonal cluster 6, 11, 20, 26, 28, 36, 38, and 39, only one clone was found. According to Table 3, some clones showed differences regarding the intensity of biofilm formation, virulence factors, and resistance to antimicrobials. The other clones had identical characteristics. The LBUEL-H204/LBUEL-H219 and LBUEL-H192/LBUEL-H244 clones, belonging to the clonal cluster 24, were isolated from BHU-22 and BHU-13, demonstrating that there are clones with 100% similarity circulating among the BHUs. Of all the cluster found, three cluster (CC8, CC13, and CC24) grouped, at least, six strains. Clonal cluster 1, 6, 11, 13, 14, 21, 24, and 39 grouped more than one strain isolated from the same BHU. Belonging to CC21, the strains LBUEL–H168 and LBUEL–H188 were isolated from the same patient (a 1-year-old boy) with a probable case of recurrent UTI. Both infections occurred in February and March 2017. The other strain isolated from the same patient (LBUEL–H219) is found in CC24 and is a clone of a strain isolated from another patient (LBUEL–H204). The infection occurred in April 2017.

**Table 2:** Different characteristics found in clonal clusters.

Clonal Cluster (CC)	Biofilm	Virulence gene	Antibiotic resistance	Resistance gene
CC1	S/VS	TV	AMP, SXT	(−)
CC2	S/VS	TV/*ucaA* neg.	AMP, SXT	*sul2*
CC3	VS	TV	NRA	(−)
CC4	VS	TV	NRA	(−)
CC5	S/VS	TV	AMP, SXT	(−)
CC6	VS	TV	NRA	(−)
CC7	S/VS	TV	NRA	(−)
CC8	S/VS	TV	NRA	(−)
CC9	S/VS	TV/*ucaA* neg.	AMP, SXT	(−)
CC10	S	TV	AMP, SXT, NAL	*sul2*
CC11	S/VS	TV	NRA	(−)
CC12	S/VS	TV	NRA	(−)
CC13	S/VS	TV	NRA	(−)
CC14	S/VS	TV/*ucaA* neg.	NRA	(−)
CC15	S/VS	TV	AMP, SXT	*sul2*
CC16	VS	TV	NRA	(−)
CC17	S/VS	TV	NRA	(−)
CC18	S/VS	TV	AMP, CEP, FEP, CRO, CXM, GEN, SXT, NAL	*sul2*
CC19	S/VS	TV/*ucaA* neg.	AMP, SXT	*sul2*
CC20	S	TV/*ucaA* neg.	AMP, SXT, GEN	*sul2*/*sul1*
CC21	S	TV	NRA	(−)
CC22	S	TV/*ucaA* neg.	AMP, GEN	(−)
CC23	S/VS	TV	NRA	(−)
CC24	S/VS	TV/*ucaA* neg.	SXT, AMP, GEN	*sul2*/*sul1*
CC25	S	TV	NRA	(−)
CC26	S/VS	TV	AMP, SXT	*sul2*
CC27	S/VS	TV	AMP, CIP, NOR, SXT, NAL	*sul2*
CC28	VS	TV/*ucaA* neg.	AMP, CEP, FEP, CRO, CXM, CIP, NOR, SXT, NAL	(−)
CC29	S/VS	TV*ucaA* neg.	NRA	(−)
CC30	VS	TV	NRA	(−)
CC31	S/VS	TV/*ucaA* neg.	SXT	(−)
CC32	VS	TV/*ucaA* neg.	SXT	(−)
CC33	VS	TV	AMP, SXT, NAL	*sul1*/*sul2*
CC34	S/VS	TV/*ucaA* neg.	AMP, CIP, GEN, NOR, SXT, NAL	*sul1*/*sul2*
CC35	VS	TV/*ucaA* neg.	NRA	(−)
CC36	VS	TV	NRA	(−)
CC37	VS	TV	AMP, SXT	*sul2*
CC38	VS	TV	NRA	(−)
CC39	S/VS	TV	AMP, GEN, AMC, CEP	(−)
CC40	S	TV	NRA	(−)

S: strong intensity of biofilm formation; VS: very strong intensity of biofilm formation; TV: Total Virulence (strains showing all virulence genes); *ucaA*neg: strains that showed all virulence genes, except for the *ucaA* gene; NRA: no resistance to antimicrobials; *sul1*: positive for *sul1* gene; *sul2*: positive for *sul2* gene; (−): negative for all resistance genes tested; AMC: Amoxicillin + Clavulanic acid; AMI: Amikacin; AMP: Ampicillin; CEP: Cephalothin; CIP: Ciprofloxacin; CRO: Ceftriaxone; CXM: Cefuroxime; FEP: Cefepime; GEN: gentamycin; NAL: nalidixic acid; NOR: norfloxacin; SXT: trimethoprim + sulfamethoxazole.

**Table 3:** Characteristics of uropathogenic *P. mirabilis* clones.

Clone	Clonal Cluster (CC)	Strains	BHU	Biofilm, virulence, and antimicrobial resistance
Clone 1	CC6	LBUEL-H264/LBUEL-H282	40/08	S/VS; TV; NRA
Clone 2	CC8	LBUEL-H231/LBUEL-H232	11/04	VS; TV; NRA
Clone 3	CC8	LBUEL-H202/LBUEL-H213/LBUEL-H216	06/26/18	VS; TV; NRA
Clone 4	CC11	LBUEL-H260/LBUEL-H261	03/11	VS; TV; NRA
Clone 5	CC13	LBUEL-H275/LBUEL-H279	01/22	S/VS; TV; NRA
Clone 6	CC13	LBUEL-H211/LBUEL-H276	28/22	S/VS; TV; NRA
Clone 7	CC13	LBUEL-H274/LBUEL-H281	26/14	S; TV; NRA
Clone 8	CC20	LBUEL-H169/LBUEL-H210	11/06	VS; TV; AMP, SXT
Clone 9	CC24	LBUEL-H204/LBUEL-H219	22/13	S/VS; TV; NRA
Clone 10	CC24	LBUEL-H192/LBUEL-H244	22/13	VS, TV; AMP, SXT
Clone 11	CC26	LBUEL-H203/LBUEL-H221	22/07	S/VS; TV; AMP, SXT
Clone 12	CC28	LBUEL-H151/LBUEL-H152	08/09	VS; *ucaA* neg.; AMP, CEP, FEP, CRO, CXM, CIP, NOR, SXT, NAL.
Clone 13	CC36	LBUEL-H234/LBUEL-H237	16/33	VS; TV; NRA
Clone 14	CC38	LBUEL-H162/LBUEL-H163	17/03	VS; TV; NRA
Clone 15	CC39	LBUEL-H156/LBUEL-H158	13/06	S/VS; TV; AMP, GEN

S: strong intensity of biofilm formation; VS: very strong intensity of biofilm formation; TV: Total Virulence (strains showing all virulence genes); *ucaA*neg: strains that showed all virulence genes, except for the *ucaA* gene; NRA: no resistance to antimicrobials; AMC: Amoxicillin + Clavulanic acid; AMI: Amikacin; AMP: Ampicillin; CEP: Cephalothin; CIP: Ciprofloxacin; CRO: ceftriaxone; CXM: cefuroxime; FEP: cefepime; GEN: gentamycin; NAL: nalidixic acid; NOR: Norfloxacin; SXT: Trimethoprim + Sulfamethoxazole.

## 4 Discussion

In this study, virulence, resistance to antimicrobials, and clonal diversity of 183 strains of *P. mirabilis* isolated from the urine of UTI patients assisted by basic health units in Londrina, Brazil, were evaluated. *P. mirabilis* is known to be an important pathogen that is related to about 1–10% of these infections [23].

According to the results of this study, the prevalence of CA-UTI having *P. mirabilis* as a causal agent is higher in women than in men. These data are in agreement with the findings obtained by Ref. [20,22]; who found the following prevalence values: 86.8% and 69.8%, respectively. This is because of anatomical characteristics: women's urethra is shorter than that of men, and perineal contamination of the urinary tract by fecal microbiota is more frequent [24].

Of the total strains evaluated in our study, 14 were isolated from children up to 1 year of age. Of these, 10 were isolated from male patients. The LBUEL–H168, LBUEL–H188, and LBUEL–H219 strains were isolated from a 1-year-old boy between February and April 2017, at three different moments when he was assisted, indicating a case of recurrent infection. The prevalence of UTIs among male children (especially up to 1 year of age) varies among different populations, probably due to factors such as circumcision, which has been associated with a reduction of the risk of UTIs [17]. In this context, it is important to mention that UTI caused by *P. mirabilis* is more prevalent in patients with structural abnormalities of the urinary tract [23]. In addition, it is known that children with urinary tract malformations have a reduced number of *E. coli* infections and a greater number of infections caused by other Gram-negative microorganisms [27].

Regarding antimicrobial resistance, Trimethoprim + Sulfatomexazole was the antibiotic combination for which the highest resistance rate was presented, followed by ampicillin. In the study by Ref. [3]; with *P. mirabilis* isolates from patients that were not hospitalized with UTIs, the analyzed strains showed a high prevalence of resistance to these antimicrobials (53.6% for trimethoprim + sulfatomexazole and 57.9% for ampicillin). In another study by Refs. [11]; resistance rates were 48.1% for ampicillin and 39.3% for trimethoprim + sulfamethoxazole.

The resistance rate for ampicillin found in this study suggests that the resistant strains produce some beta-lactamase enzyme (99.5% of the strains were sensitive to amoxicillin + clavulanic acid and 100% to piperacillin + tazobactam). According to our statistical analysis, women aged 16–31, 48–60, and 80–95 years are 11.1 (p < 0.001; CI: 2.81–43.76), 6.91 (p < 0.001; CI: 2.2–21.68), and (p = 0.024; CI: 1.15–9.15) times more likely to have *P. mirabilis* infection with ampicillin resistance, respectively.

A study carried out in France with patients from the community and from hospitals showed that *P. mirabilis* was the second most frequent microorganism (5.2%) in the urine of male patients who used a catheter and presented high resistance rates for ampicillin (40%), amoxicillin (40%), ticarcillin (42%), gentamicin (18%), nalidixic acid (26%), norfloxacin (22%), ciprofloxacin (21%), trimethoprim + sulfamethoxazole (32%), phosphomycin (19%), and nitrofurantoin (100%) [13].

A study in the Kinki region (Japan) demonstrated that the isolation rates of ESBL-producing *P. mirabilis* increased from 0% to 12.9% in the period from 2000 to 2009 [25]. [4] conducted a study with 8836 clinical isolates of Enterobacteria. Of the total strains producing ESBL, 5.2% were *P. mirabilis*, and *CTX-M-1* and *CTX-M-9* were the predominant genes found. In our study, only one strain produced ESBL, which contained the *CTX-M-9* phenotype.

In this study, strains that presented a resistance phenotype to, at least, one antimicrobial of the class of quinolones, sulfonamides, and/or aminoglycosides, were subjected to PCR to assess the presence of some resistance genes. Of the 40 strains resistant to trimethoprim + sulfamethoxazole, 7 (17.5%) were positive for *sul1* and 20 (50%) were positive for *sul2*; 4 (10%) strains had both genes. The other analyzed genes were not found in any of the tested isolates. These results indicate that resistance to quinolones and aminoglycosides may be related to other genes that were not investigated, as well as to strains that are resistant to sulfonamide Trimethoprim + Sulfamethoxazole, which did not contain the *sul1* or *sul2* gene.

*P. mirabilis* is among the most common pathogens of urinary tract infection; cystitis and acute pyelonephritis are common types of UTI caused by this species. The severity of UTI caused by *P. mirabilis* depends on the expression of several virulence factors, biofilm formation ability, and resistance to the different classes of antimicrobials used against these pathogens [23]. Our results indicate a high prevalence of virulence factors among the studied isolates [23]. conducted a study on the virulence profile of *P. mirabilis* strains isolated from patients with UTI and found that 100% of the strains were positive for the *mrpA*, *pmfA,* and *hmpA* genes, whereas the *zapA*, *ptA,* and *ucaA* genes were found in 98.2%, 95.5%, and 95.5% of the strains, respectively [15]. also found high rates in their research: 98.4% of the strains were positive for the *hpmA* and *atfA* genes, and 92.1%, for the *mrpA* gene. These results, together with ours, show the high prevalence rates of virulence genes found in uropathogenic *P. mirabilis* strains.

In the present study, all strains tested were capable of forming biofilms at different intensities. These results are in agreement with the studies carried out by Refs. [16,28]; who only found biofilm-producing strains. The high biofilm formation rates found here serve as a warning, since these structures are capable of blocking urinary catheters and to difficult the antibiotic action and the immune response of the host.

Regarding genetic diversity, forty different clonal cluster were found. Some of these clusters harbored strains with the same virulence and resistance (genotypic and phenotypic) characteristics, while others harbored strains with different characteristics. This suggests that some characteristics may have been acquired through the acquisition of plasmids, via mutations or through other unknown mechanisms. All clones found differed according to BHU and, consequently, to the location from which they were isolated, suggesting the circulation of clonal uropathogenic *P. mirabilis* strains in the city of Londrina.

A study by Ref. [31] with *P. mirabilis* isolates from different sources (UTI, wound, respiratory secretion, meat, and isolates from other animals) revealed that there were no clonal relationships (using the ERIC-PCR technique) among the strains studied. In addition, they demonstrated that clinical and non-clinical isolates showed a similar ability to cause UTI in mice, concluding that, regardless of the origin and genetic diversity, *P. mirabilis* is commonly characterized as an important causative pathogen of urinary tract infections.

In general, our findings show that *P. mirabilis* strains isolated from CA-UTI patients in the city of Londrina can be divided into several clonal clusters. In addition, we observed that there may be genetically related strains circulating in the same BHU, and strain clones circulating in the city.

Our results showed that *P. mirabilis* uropathogenic strains isolated from BHUs have several virulence characteristics, including the production of cytotoxins, fimbriae, and iron acquisition systems, which are important pathogens that cause UTIs in the community. The high capacity of this pathogen to form biofilms is a worrying factor in the case of a complicated infection, as this makes antibiotic therapy difficult. The sensitivity profile to antimicrobials revealed that some *P. mirabilis* strains have resistance to antibiotics that are commonly used in the treatment of UTIs. In addition, it was observed that there were clones of *P. mirabilis* circulating in the city of Londrina, and genetically similar strains in the BHUs evaluated in this study.

## CRediT authorship contribution statement

**Wellington Danilo de Oliveira:** Investigation, Validation. **Mário Gabriel Lopes Barboza:** Investigation. **Gabriela Faustino:** Investigation. **Willian Teruhiko Yamanaka Inagaki:** Investigation. **Matheus Silva Sanches:** Investigation, Methodology, Validation. **Renata Katsuko Takayama Kobayashi:** Resources, Formal analysis. **Eliana Carolina Vespero:** Funding acquisition, Data curation. **Sergio Paulo Dejato Rocha:** Conceptualization, Writing - review & editing, Project administration, Supervision.

## Declaration of competing interest

The authors declare that they have no conflicts of interest.

## Figures and Tables

**figs1:**